# Risk assessment in interstitial lung disease: the incremental prognostic value of cardiopulmonary ultrasound

**DOI:** 10.1186/s12890-021-01606-3

**Published:** 2021-07-15

**Authors:** Wei-wei Zhu, Hong Li, Yi-dan Li, Lanlan Sun, Lingyun Kong, Xiaoguang Ye, Qizhe Cai, Xiu-zhang Lv

**Affiliations:** Department of Echocardiography, Beijing Chao Yang Hospital, Capital Medical University, No. 8 Gongren Tiyuchang Nanlu, Chao Yang District, Beijing, 100020 China

**Keywords:** Cardiopulmonary ultrasound, Interstitial lung disease, Pulmonary fibrosis, ILD-GAP score

## Abstract

**Background:**

The mortality risk of chronic interstitial lung disease (ILD) is currently assessed using the ILD-GAP score. The present study evaluates whether the addition of cardiopulmonary ultrasound parameters to the ILD-GAP score can further improve the predictive value of ILD-GAP.

**Methods:**

Medical records from 91 patients with ILD hospitalized from June 2015 to March 2016 were retrospectively examined. The Lung ultrasound (LUS) score, right ventricular (RV) function, and mechanics were obtained from the cardiopulmonary ultrasound. The ILD-GAP score was calculated from demographic characteristics and pulmonary function parameters. Patients were followed up with until May 2020. The primary endpoint was all-cause death.

**Results:**

After exclusions, 74 patients with ILD were included in the analysis. During the follow-up period, 36 patients with ILD survived (ILD_s_), and 38 patients died (ILD_d_). Compared to ILD_s_, the ILD_d_ cases exhibited a higher number of B-lines, LUS score, and RV end-diastolic base dimension (RVD), but lower RV function. In multivariate analysis, the ILD-GAP score (hazard ratio, 2.88; 95% CI 1.38–5.99, *P* = 0.005), LUS score (hazard ratio 1.13; 95% CI 1.04–1.24, *P* = 0.006), and RVD (hazard ratio 1.09, 95% CI 1.03–1.16, *P* = 0.004) were significantly related to the risk of death. Adding the LUS score and RVD to the ILD-GAP score significantly improved the predictive value compared to the ILD-GAP score alone (C statistics 0.90 vs 0.76, *P* = 0.018).

**Conclusion:**

We investigated the utility of a new prognostic model for ILD that includes both cardiopulmonary ultrasound parameters (LUS score and RVD) and the ILD-GAP score. This model better reflects the severity of pulmonary fibrosis and cardiac involvement, and has incremental predictive value over the ILD-GAP score alone.

## Background

Interstitial lung disease (ILD) is an umbrella term used to describe a large group of heterogenous pulmonary diseases characterized by chronic inflammation and fibrosis of the lung parenchyma [1]. The heterogeneity of ILD makes it difficult to reliably predict the prognosis of the disease. Ryerson et al. developed a modified gender-age-physiology (GAP) model—the ILD-GAP model that has been shown to reliably predict disease severity and survival based on gender, age, ILD subtype and lung physiology parameters [Forced Vital Capacity (%FVC) and diffusing capacity of the lung for carbon monoxide (%DLCO)] [2]. Nevertheless, the ILD-GAP score is a simple prediction model that does not take into account lung morphology and cardiac involvement.

Chest high-resolution computed tomography (HRCT) is the gold-standard imaging technique in the assessment of ILD [3]. However, exposure to ionizing radiation and high cost limit the use of HRCT, especially for regular follow-ups. In the past decade, lung ultrasound (LUS) has emerged as a tool for the assessment of ILD by detecting and quantifying sonographic B-lines and pleural line irregularities [4–7]. Studies have demonstrated that LUS exhibits good diagnostic accuracy for ILD, and the results have correlated well with HRCT or chest X-ray findings [8, 9]. A scoring system for the evaluation of pulmonary fibrosis in patients with ILD based on LUS findings was proposed by Buda et al. [10], the results of which correlated well with the HRCT Warrick score. Our team’s previous study showed a significant correlation between a LUS score based on multiple LUS signs and systolic pulmonary artery pressure (SPAP) obtained by echocardiography in patients with ILD [11]. LUS and echocardiography have different but complementary features, and their combined use can effectively evaluate cardiopulmonary function. The recent European Society of Cardiology (ESC) expert consensus statement suggested that an integrative approach that includes both echocardiography and LUS should be recommended for the assessment and management of acute heart failure [12]. For patients with ILD, cardiopulmonary ultrasound, which is the combined use of LUS and echocardiography, can not only assess the severity of pulmonary fibrosis but also cardiac involvement, such as pulmonary hypertension or right heart failure, which is highly correlated with a poorer prognosis. This allows the most appropriate, individualized interventions to be delivered to the patients [13, 14].

However, the prognostic value of cardiopulmonary ultrasound in patients with ILD, and whether it can add value to the ILD-GAP score, is still unknown. This study aimed to assess the added value of using cardiopulmonary ultrasound parameters in the risk assessment of patients with ILD.

## Methods

### Study population

We designed a single-center, retrospective study that included 91 hospitalized patients with ILD. All patients were treated in the respiratory department of Beijing Chaoyang Hospital Affiliated to Capital Medical University between June 2015 and March 2016 and underwent cardiopulmonary ultrasound, pulmonary function tests (PFTs), and HRCT during hospitalization. All baseline data were acquired based on a review of the patients’ charts. Routine follow-ups were performed until the end of May 2020.

The exclusion criteria were as follows: lung transplantation during the follow-up period (n = 3), history of pulmonary surgery (n = 2), respiratory tract infections, another underlying lung disease such as chronic obstructive pulmonary disease (n = 2), severe left ventricular failure (n = 2), poor echocardiographic image quality (n = 4) and those with missing follow-up information (n = 4). Furthermore, echocardiography was performed in all cases in this study to identify and rule out patients with valvular heart disease. Patients with a history of coronary heart disease, atrial fibrillation and lung cancer were also excluded. Following exclusions, 74 patients with ILD remained for further analysis.

The ILD diagnosis was made following a multidisciplinary review of clinical, radiological, and pathological data [1, 3, 15]. ILD cases were further divided into the following subtypes: idiopathic pulmonary fibrosis (IPF), idiopathic nonspecific interstitial pneumonia, connective tissue disease-associated ILD, chronic hypersensitivity pneumonitis, and unclassifiable ILD.

The study protocol was approved by the institutional review board of Beijing Chaoyang Hospital Affiliated to Capital Medical University. Informed consent was waived due to the retrospective nature of the study. Data from some patients was also used in a previous study.

### Cardiopulmonary ultrasound

The acquisition scheme of cardiopulmonary ultrasound used in the current work is consistent with that of a previous study from our team [11]. Commercially available echocardiographic equipment with a 1–5 MHz cardiac sector transducer and a 3–11 MHz linear transducer was used (IE33, Philips Medical Systems, Andover, MA, USA) to acquire cardiopulmonary ultrasound images. The total number of B-lines and a semi-quantitative LUS score were used to assess the severity of pulmonary fibrosis [10]. Two-dimensional and Doppler echocardiographic parameters were measured, including left ventricular end-diastolic diameter, left ventricular ejection fraction, right ventricular end-diastolic base dimension (RVD), primary pulmonary artery diameter, tricuspid annular plane systolic excursion (TAPSE), right ventricular fractional area change (RVFAC), peak systolic tricuspid annulus velocity, myocardial performance index, and SPAP as estimated by tricuspid regurgitation. The right ventricular global longitudinal strain (RVGLS) was automatically tracked using the Philips QLAB Version 13.0 software (Philips Healthcare). Two experienced lung ultrasound physicians (Dr. Weiwei Zhu and Dr. Hong Li) performed intra- and inter-observer repeatability analyses of all patients’ LUS scores in 2-day intervals. The observers were blind to the clinical status, HRCT, PFTs, and all patients’ medical information.

### Clinical outcome

Patients were followed up with at our outpatient clinic by clinical visits and telephone calls until the end of May 2020. The primary endpoint was the composite of the all-cause death. For patients without events, the date of the last contact was used for survival analysis.

### Statistical analysis

Statistical analysis was performed using SPSS Version 20 for Windows (IBM, Armonk, NY, USA) and the MedCalc Version 15.6.1 (MedCalc Software, Ostend, Belgium) statistical software. Continuous variables are expressed as mean ± standard deviation or median and interquartile ranges, where appropriate. Categorical data are presented as absolute numbers and percentages. Two-sample comparisons were performed using a *t* test if variables were normally distributed, or a Mann–Whitney U-test if variables were not normally distributed. Categorical data was compared using a chi-squared test or a Fisher’s exact test. Univariate and multivariate logistic regression techniques were used to identify the independent risk factors related to the prognosis of patients with ILD. The cutoff values for the ILD-GAP score, LUS score, RVD, and RVGLS were determined with a receiver operating characteristic (ROC) curve analysis and a Delong test for ROC curve comparison. Survival was estimated using the Kaplan–Meier method. A log-rank test was used to compare the survival rate between the two groups. To determine the independent risk factors of survival, a Cox proportional-hazards model was used. We employed a sequential Cox model method to determine the incremental value of cardiopulmonary ultrasound parameters over the ILD-GAP score alone, in association with the primary endpoint. Specifically, the incremental value was defined by a significant increase in the C statistic value.

## Results

### Baseline characteristics

During the follow-up period (median 50.0 months, interquartile range 14.8–54.3 months), 38 patients died (ILD_d_), and 36 patients survived (ILD_s_). The baseline characteristics of the study population, divided according to survival status, is shown in Table 1. There were no significant differences in the distribution of ILD subtypes between the two groups. The mean age was significantly higher in ILD_d_ compared to ILD_s_, and the percentage of women was also lower. Arterial oxygen partial pressure (PaO_2_), arterial oxygen saturation (SaPO_2_), FVC%, DLCO%, and total lung capacity (TLC%) were all lower in ILD_d_ compared to ILD_s_, whereas the ILD-GAP score was higher.

**Table 1:** Baseline characteristics

Variable	Total (N = 74)	ILD_s_ (N = 36)	ILD_d_ (N = 38)	*P* value
Age (years)	59.47 ± 9.63	55.72 ± 9.94	63.00 ± 8.00	0.001
Women, n (%)	39.19%	55.56%	23.68%	0.005
Duration of disease (month)	12 (2.8–48)	6 (1–12)	48 (12–72)	< 0.001
ILD subtypes, n (%)				
IPF	14 (18.9%)	6 (16.7%)	8 (21.1%)	
Unclassifiable ILD	26 (35.1%)	13 (36.1%)	13 (34.2%)	
CTD-ILD	23 (31.1%)	10 (27.8%)	13 (34.2%)	
Idiopathic NSIP	6 (8.1%)	3 (8.3%)	3 (7.9%)	
HP	5 (6.8%)	4 (11.1%)	1 (2.6%)	
SBP (mmHg)	127.55 ± 14.81	129.33 ± 13.54	125.87 ± 15.91	0.318
DBP (mmHg)	77.77 ± 9.82	77.92 ± 9.21	77.63 ± 10.49	0.902
PaO_2_ (mmHg)	75.78 ± 16.04	79.64 ± 14.35	71.81 ± 16.91	0.039
PaCO_2_ (mmHg)	41.52 ± 7.37	41.5 (39.25–44.00)	39.91 ± 5.68	0.092
SaPO_2_ (%)	96 (91–97)	96 (94–97)	95 (90–96)	0.034
NT-proBNP (pg/ml)	50.56 (33.40–219.42)	33.40 (23.74–47.96)	125.00 (50.40–590.87)	< 0.001
FVC%	77.86 ± 18.81	83.89 ± 16.58	71.1 ± 19.16	0.012
DLCO%	51.74 ± 18.80	59.52 ± 16.71	43.06 ± 17.50	0.001
TLC%	70.52 ± 17.12	77.20 ± 17.19	63.04 ± 13.89	0.002
ILD-GAP score	2 (0–3)	1 (-1–2)	3 (1–4)	< 0.001

Data are presented as mean ± SD (normally distributed) or median [IQR] (not normally distributed)

*ILD* interstitial lung disease, *IPF* idiopathic pulmonary fibrosis, *CTD* connective tissue disease, *NSIP* nonspecific interstitial pneumonia, *HP* hypersensitivity pneumonitis, *SBP* systolic blood pressure, *DBP* diastolic blood pressure, *PaO*_*2*_ arterial oxygen partial pressure, *PaCO*_*2*_ arterial carbon dioxide partial pressure, *SaPO*_*2*_ arterial oxygen saturation, *NT-proBNP* N-terminal pro brain natriuretic peptide, *FVC* forced vital capacity, *DLCO* diffusing capacity of the lung for carbon monoxide, *TLC* total lung capacity, *GAP* gender, age, physiology

### Cardiopulmonary ultrasound parameters

Several cardiopulmonary ultrasound parameters differed significantly between ILD_d_ and ILD_s_ patients (Table 2). The ILD_d_ group exhibited a larger RVD and primary pulmonary artery diameter (D_MPA_), and a lower RV function than the ILD_s_ group. The RV function was characterized by the TAPSE, RVFAC and RVGLS. The mean RVGLS value of the ILD_d_ group was even lower than the normal range recommended by the accepted guidelines (− 18.6% vs − 20.0%) [16]. ILD_d_ patients had significantly more severe pulmonary fibrosis, as shown by both the higher total number of B-lines and the LUS score. The intra- and inter-observer interclass correlation coefficients for the LUS score were 0.90 (0.84–0.94) and 0.84 (0.76–0.90), respectively. The mean intra- and inter-observer differences in the LUS score were − 0.20 ± 2.19 (limits of agreement, − 4.5 to 4.1) and − 0.6 ± 2.63 (limits of agreement, − 5.8 to 4.5), respectively.

**Table 2:** Cardiopulmonary ultrasound parameters

Variable	Total (N = 74)	ILD_s_ (N = 36)	ILD_d_ (N = 38)	*P* value
LVEDD (mm)	44.07 ± 3.53	44.03 ± 2.90	44.12 ± 4.08	0.912
LVEF (%)	69.50 ± 4.51	69.20 ± 4.51	69.78 ± 4.56	0.581
RVD (mm)	33.90 ± 5.70	30.98 ± 2.99	36.67 ± 6.28	< 0.001
D_MPA_ (mm)	24.85 ± 3.21	23.68 ± 2.16	25.95 ± 3.66	0.002
TAPSE (mm)	19.90 ± 2.75	20.19 ± 2.19	19.61 ± 3.20	0.361
RVFAC (%)	43.26 ± 6.78	45.81 ± 4.04	40.85 ± 7.93	0.001
S′ (cm/s)	13.04 ± 2.66	12.50 ± 2.25	13.55 ± 2.93	0.090
MPI	0.56 ± 0.144	0.50 ± 0.11	0.61 ± 0.16	0.002
SPAP (mmHg)	29.50 (25.52–38.70)	27.61 ± 4.42	43.23 ± 19.36	< 0.001
RVGLS (%)	− 21.50 (− 22.90 to − 19.25)	− 22.33 ± 2.96	− 18.60 ± 4.64	< 0.001
B lines	44.45 ± 18.66	35.42 ± 15.99	53 ± 17.06	< 0.001
LUS score	15.45 ± 4.72	13.06 ± 3.93	17.71 ± 4.30	< 0.001

Data are presented as mean ± SD or median [IQR] when data is not normally distributed

*LVEDD* left ventricular end-diastolic diameter, *LVEF* left ventricular ejection fraction, *RVD* right ventricular end-diastolic base dimension, *D*_*MPA*_ primary pulmonary artery diameter, *TAPSE* tricuspid annular plane systolic excursion, *RVFAC* right ventricular fractional area change, *S*′ peak systolic tricuspid annulus velocity, *MPI* myocardial performance index, *SPAP* systolic pulmonary artery pressure, *RVGLS* right ventricular global longitudinal strain, *LUS* lung ultrasound

### The prognostic value of cardiopulmonary ultrasound parameters

**Fig. 1:**
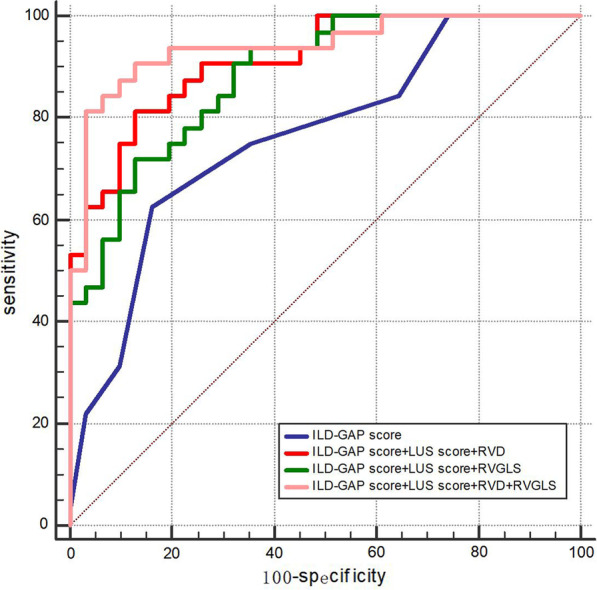
Receiver operating characteristic (ROC) analysis of the incremental association value of cardiopulmonary parameters when added to the ILD-GAP score. Model 1: ILD-GAP score, AUC = 0.76; Model 2: ILD-GAP score + LUS score + RVD, AUC = 0.91; Model 3: ILD-GAP score + LUS score + RVGLS, AUC = 0.88; Model 4: ILD-GAP score + LUS score + RVD + RVGLS, AUC = 0.94. Model 1 versus Model 2 Comparison *P* value = 0.005; Model 1 versus Model 3 Comparison *P* value = 0.033; Model 2 versus Model 3 Comparison *P* value = 0.440; Model 3 versus Model 4 Comparison *P* value = 0.069

Univariate and multivariate logistic regression analysis showed that the ILD-GAP score, RVD, RVGLS, and LUS score were independently correlated with poor prognosis in patients with ILD (Table 3). Receiver operating characteristic (ROC) analysis of the incremental value of combining cardiopulmonary ultrasound parameters with the ILD-GAP score for risk assessment revealed two optimal models. The first was model 2, which consisted of the ILD-GAP score, LUS score, and RVD (C statistics 0.91 vs 0.76, *P* = 0.005), and the second was model 3, which consisted of the ILD-GAP score, LUS score and RVGLS (C statistics 0.88 vs 0.76, *P* = 0.033) (Fig. 1). The C statistic values between the two models were not significantly different (0.91 vs 0.88, *P* = 0.44).

**Table 3:** Univariate and multivariate logistic regression analysis for predictors of the risk of death in patients with ILD

	Univariable (OR, 95% CI)	*P* value	Multivariable (OR, 95% CI)	*P* value
ILD-GAP score	1.77 (1.27–2.46)	0.001	2.25 (1.29–3.92)	0.004
RVD (mm)	1.37 (1.15–1.63)	< 0.001	1.66 (1.20–2.29)	0.002
SPAP (mmHg)	1.14 (1.05–1.23)	0.001		
RVFAC (%)	0.87 (0.79–0.96)	0.003		
RVGLS (%)	1.36 (1.13–1.65)	0.002	1.54 (1.11–2.14)	0.010
B lines	1.07 (1.03–1.11)	< 0.001		
LUS score	1.30 (1.12–1.50)	< 0.001	1.32 (1.02–1.70)	0.034

*OR* odds ratio, *CI* confidence interval, *ILD-GAP* interstitial lung disease-gender, age, physiology, *RVD* right ventricular end-diastolic base dimension, *RVFAC* right ventricular fractional area change, *SPAP* systolic pulmonary artery pressure, *RVGLS* right ventricular global longitudinal strain, *LUS* lung ultrasound

**Fig. 2:**
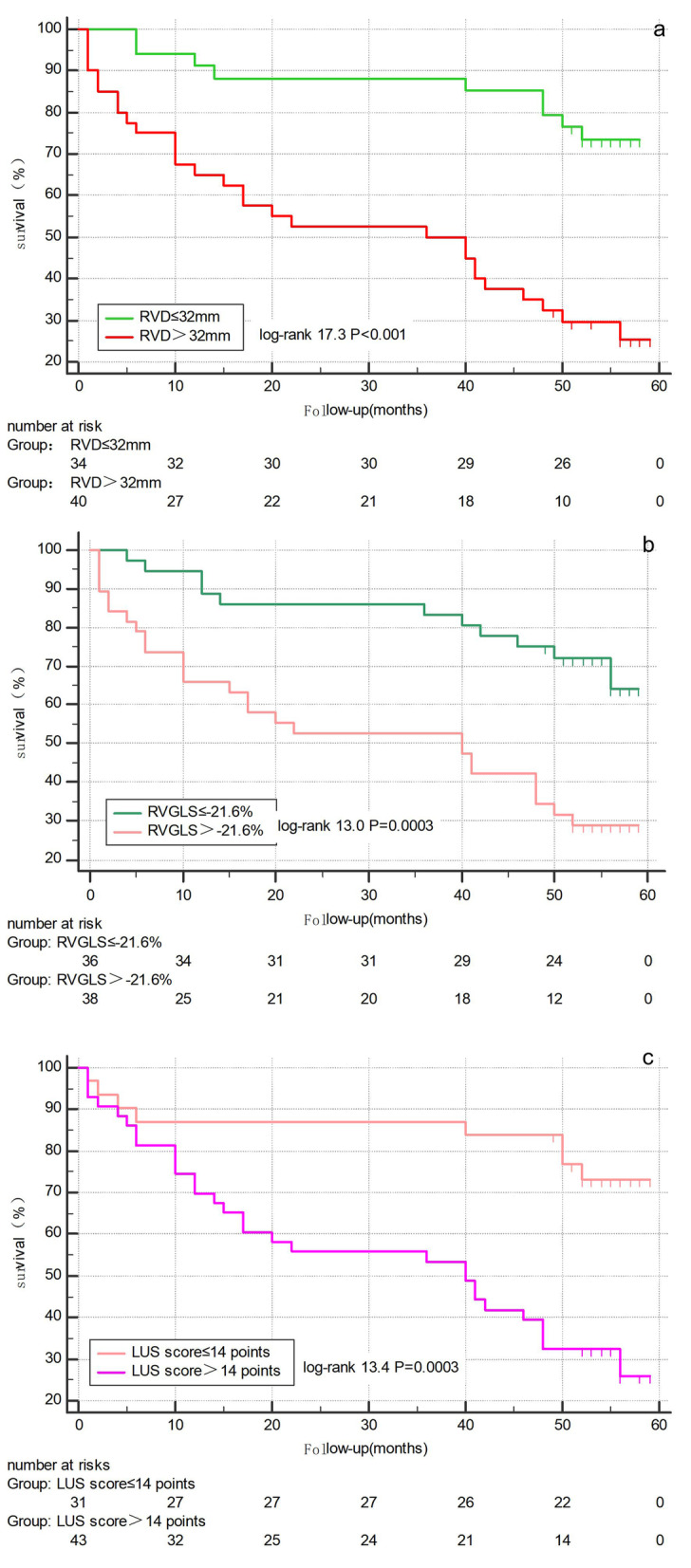
Kaplan–Meier analysis of event-free survival. Patients were stratified according to the cutoff value of the RVD, RVGLS, and LUS score. **a** RVD > 32 mm and RVD ≤ 32 mm for patients with ILD. **b** RVGLS > − 21.6% and RVGLS ≤ − 21.6% for patients with ILD. **c** LUS score > 14 points and LUS score ≤ 14 points for patients with ILD

The cutoff values of the RVD, RVGLS, and LUS score associated with risk of death in patients with ILD were determined using the ROC analysis. The resulting cutoff values were 32 mm for RVD, − 21.6% for RVGLS and 14 points for LUS score. ILD patients with a larger RVD (> 32 mm), a worse RVGLS (> − 21.6%) and a higher LUS score (> 14 points) had a significantly worse chance of survival (Fig. 2).

**Fig. 3:**
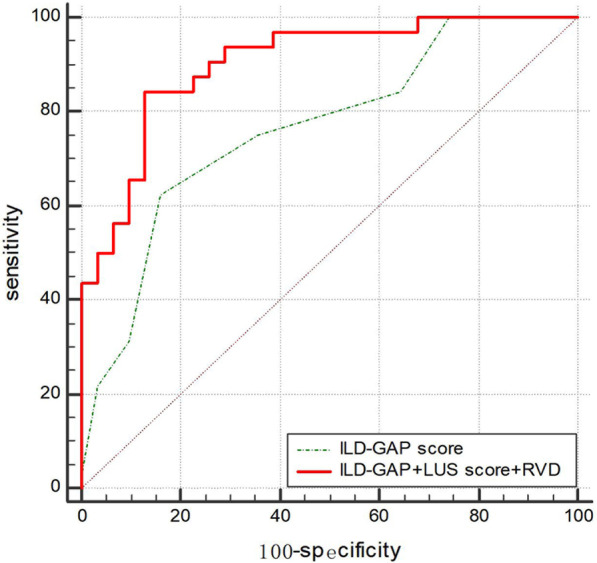
Receiver operating characteristic (ROC) analysis of the incremental association value of cardiopulmonary ultrasound (LUS score and RVD) when added to the ILD-GAP score. Model 1: ILD-GAP score, AUC = 0.76; Model 2: ILD-GAP score + LUS score + RVD, AUC = 0.90; Model 1 versus Model 2 Comparison *P* value = 0.018

To further determine the effect of cardiopulmonary ultrasound parameters on the survival time of patients with ILD, we performed univariate and multivariate Cox proportional-hazards regression analyses (Table 4). The primary outcome (all-cause mortality) was associated with the ILD-GAP score, RVD, RVGLS, and LUS score in the univariate model. A stepwise multiple regression model revealed that the ILD-GAP score, RVD, and LUS score were all correlated with the prognosis of patients with ILD, but not the RVGLS. A direct comparison of the prediction value of these three parameters was made, the result of which showed that there was no statistically significant difference between these three parameters in the current cohort. The C statistics of the ILD-GAP score, the LUS score, and the RVD were 0.76, 0.76 and 0.79, respectively. We further used C statistics to assess the effects of combining several cardiopulmonary parameters with the ILD-GAP score by comparing model 1 (ILD-GAP score only) with model 2 (model 1 + LUS score + RVD). The cardiopulmonary ultrasound parameters showed incremental prognostic value over the ILD-GAP score alone [C statistics 0.90 vs 0.76, *P* = 0.018 (Fig. 3)].

**Table 4:** Univariate and multivariate Cox proportional-hazards regression analysis for the association with the risk of death in patients with ILD

	Univariable (HR, 95% CI)	*P* value	Multivariable (HR, 95% CI)	*P* value
ILD-GAP score	3.80 (1.27–2.46)	< 0.001	2.88 (1.38–5.99)	0.005
RVD (mm)	1.16 (1.10–1.23)	< 0.001	1.09 (1.03–1.16)	0.004
RVGLS (%)	1.18 (1.10–1.26)	< 0.001		
LUS score	1.18 (1.10–1.27)	< 0.001	1.13 (1.04–1.24)	0.006

*HR* hazard ratio, *CI* confidence interval, *ILD-GAP* interstitial lung disease-gender, age, physiology, *RVD* right ventricular end-diastolic base dimension, *RVGLS* right ventricular global longitudinal strain, *LUS* lung ultrasound

## Discussion

The main finding of the present study is that the combination of cardiopulmonary ultrasound parameters, notably the LUS score and RVD, with the ILD-GAP score improves the prediction of prognosis in patients with ILD compared to the ILD-GAP score alone. This shows that cardiopulmonary ultrasound is a useful method that aids risk stratification in patients with ILD. Aside from the severity of pulmonary fibrosis, the cardiac impact of ILD—right ventricular remodeling and dysfunction are also important in the prognosis of ILD.

### RV remodeling and dysfunction correlated with poor outcome in patients with ILD

Pulmonary hypertension (PH) is a frequent and severe complication in IPF and other ILDs [17]. Pulmonary fibrosis-induced destruction of pulmonary vessels, hypoxic pulmonary vasoconstriction and pulmonary vascular remodeling induced by vasoactive compounds can all lead to PH [18]. The RV is highly sensitive to pressure overload [19] and the gradual increase of SPAP in patients with ILD causes compensatory enlargement and hypertrophy of the RV, which can lead to dysfunction, right heart failure and death.

Using multivariate analysis, we showed that RVD and RVGLS were more related to the outcome of patients with ILD than the SPAP parameter. This finding is in agreement with the study on IPF patients by Rivera-Lebron et al. [20] and other previous studies that examined PH [21]. Andersen et al. [13] showed that ILD patients with PH had markedly higher mortality than those without PH. Our study also found that the death group had a higher proportion of PH cases, but no significant correlation between SPAP and prognosis.

In our study, only RVGLS was found to correlate with the prognosis of patients with ILD, but not the traditional right ventricular functional parameters (i.e. TAPSE, RVFAC). This might be because such parameters do not show abnormalities in the early stage of right ventricular remodeling. Nevertheless, a previous study showed that RVGLS could reveal early impairment of right ventricular function, even in IPF patients without pulmonary hypertension [22].

Although this study initially found a correlation between RVGLS and the risk of death in patients with ILD, this correlation was not confirmed by the multivariate Cox proportional hazard regression model. This might be due to the fact that right ventricular function was relatively preserved among the patients enrolled in the present study. The median value of the RVGLS was − 21.5%, which was significantly higher than the − 20% value that is recommended by the ASE guidelines [16]. This may be the reason why RVGLS was not found to be related to the prognosis of patients with ILD in our multivariate analysis.

The RV parameter that correlated with prognosis in patients with ILD was RVD. Bax et al. [23] showed in a recent study that the ratio between the RV and the left ventricle on CT pulmonary angiograms predicted mortality in ILD, rather than echocardiography parameters such as TAPSE or RVFAC. These findings demonstrated the importance of morphological changes in the RV, especially when SPAP cannot be obtained. A change in RV shape and volume can be the first sign of RV dysfunction or volume overload [24].

### ILD LUS score correlated with poor outcome in patients with ILD

A large number of previous studies have demonstrated the accuracy of LUS in evaluating the severity of pulmonary fibrosis in patients with ILD [6–8, 25]. Severe lesions of ILD are often present in the peripheral area of the subpleural lung, which facilitates the evaluation of the severity of pulmonary fibrosis by LUS [26]. Previous studies conducted by our team revealed a certain correlation between the LUS score and PFT parameters (DLCO%, FVC%), the ILD-GAP score, and even some echocardiographic parameters, including SPAP and RVGLS, in patients with ILD [11, 27]. The present study further found the LUS score to be an independent predictor of death in patients with ILD.

LUS has been shown to be useful in the evaluation of all stages of ILD. In the early stage of ILD, when pulmonary parenchyma injury and alveolitis manifests, as well as when increased fibrous components in the alveolar septum, thickened alveolar wall, and interlobular septal are observed, numerous B-line artifacts or white lung indicators can be detected by LUS when the subpleural lung tissue is involved. When the progressive aggravation of alveolar inflammatory injury leads to the destruction of the alveolar wall, and the appearance of subpleural cysts and a honeycomb, LUS will show blurred pleura lines [1, 10]. Recently, Gargani et al. analyzed the relationship between B lines and prognosis in patients with systemic sclerosis. Their results showed that B lines were an independent predictor of ILD deterioration [28]. Although their study ignored the importance of pleural lines in evaluating lung involvement, they still found a predictive value of B-lines. All of these findings indicate that LUS is a useful tool for evaluating lung pathology in patients with ILD.

### Clinical implications

The ILD-GAP score currently used for risk assessment of ILD patients largely depends on the FVC and DLCO. Prior to disease development, the normal pulmonary function of a person can range from 80 to 120% of values predicted from age, sex and height. A FVC threshold of 75% would therefore represent a decline of between 5 and 45% depending on premorbid values, a ninefold variability. It is therefore necessary to use HRCT to refine the assessment and identify patients in whom measured values are misleading [29]. However, HRCT is not feasible for dynamic monitoring in the intensive care unit or for regular follow-up sessions in chronic patients. LUS on the other hand, is much more suitable for dynamic monitoring and can be easily repeated without exposing patients to ionizing radiation.

In clinical practice, close attention is paid to lung lesions in patients with ILD, but their right ventricular remodeling is largely ignored. In our cohort study, we found that RVD can add additional predictive value to the ILD-GAP score. RVD is also an easily obtainable parameter. Although we failed to obtain RVGLS in some patients, we found that using RVGLS in combination with RVD could not improve prediction efficiency. When evaluating the RV in patients with ILD, if RVGLS cannot be obtained, the dilatation of RVD is considered a sign of poor prognosis. Therefore, the addition of the two cardiopulmonary ultrasound parameters (LUS score and RVD) to the ILD-GAP score may help to further refine the risk stratification of patients with ILD.

The inclusion of a cardiopulmonary ultrasound in the work up requires extra resources, both human and financial, but can also decrease the burden of physicians and patients by reducing the need for HRCT. The acquisition of the LUS score and RVD is simple and quick, requiring less than 5 min to perform even when both LUS and echocardiography are included. The LUS is therefore useful for daily monitoring of ILD patients in a hectic hospital setting, whether in the intensive care unit, in the general ward or in the outpatient clinic. Compared with the high cost of HRCT examination, the application of ultrasound greatly facilitates clinical management and reduces the medical expenses for patients.

### Limitations

Various limitations of the current work should be noted. This study is a retrospective single-center study with a small sample size, which limits the generalizability of the conclusions drawn. The endpoint occurrence (all-cause death) was determined by telephone follow-up in almost all cases, which can be inaccurate due to recall bias of the patients’ families. A large-scale prospective multicenter study is needed to confirm the results of this work in the future.

Standardized methods for evaluating patients with ILD by LUS are lacking, both for pulmonary fibrosis, and RV function and structure. The present study used the LUS score, which was developed by Buda et al. to evaluate pulmonary fibrosis [10]. For RV function, previous studies have shown that RVGLS might be a sensitive parameter [16]. However, since no three-dimensional echocardiographic images were collected in the current work, we were unable to evaluate the correlation between RV volume [24] and prognosis of patients with ILD. Nevertheless, we still found a good correlation between the structural parameter RVD and the risk of death in patients with ILD.

This study only focused on whether the addition of cardiopulmonary ultrasound parameters could increase the prediction value of the ILD-GAP score. There are also other scores that have been utilized in the survival prediction of IPF patients, such as the TORVAN index [30]. It would be interesting to investigate whether the addition of the cardiopulmonary ultrasound parameters could also increase the prediction value of the other scores as well.

In all, this study preliminarily explored the prognostic value of cardiopulmonary ultrasound parameters in patients with ILD. This question has been rarely studied in the past, and based on the current and encouraging results, we will perform large-scale prospective studies in the future.

## Conclusions

Cardiopulmonary ultrasound is a useful tool for assessing the risk of death in patients with ILD. The addition of cardiopulmonary ultrasound parameters, such as LUS score and RVD, to the ILD-GAP score can better identify patients with worse prognosis and who are in need of a closer clinical-therapeutic surveillance. Cardiopulmonary ultrasound may be incorporated into the routine management of patients with ILD in the future, especially in critically ill patients.

## Data Availability

All data supporting the findings of this study are available within the manuscript. The datasets used and/or analysed during the current study are available from the corresponding author on reasonable request.

## Abbreviations

DLCO: Diffusing capacity of the lung for carbon monoxide
FVC: Forced vital capacity
GAP: Gender, age, physiology
HRCT: High-resolution computed tomography
ILD: Interstitial lung disease
IPF: Idiopathic pulmonary fibrosis
LUS: Lung ultrasound
PaO_2_: Arterial oxygen partial pressure
PFTs: Pulmonary function tests
PH: Pulmonary hypertension
ROC: Receiver operating characteristic
RVD: Right ventricular end-diastolic base dimension
RVFAC: Right ventricular fractional area change
RVGLS: Right ventricular global longitudinal strain
SaPO_2_: Arterial oxygen saturation
SPAP: Systolic pulmonary artery pressure
TAPSE: Tricuspid annular plane systolic excursion

## References

[1] Wallis A, Spinks K (2015). The diagnosis and management of interstitial lung diseases. BMJ.

[2] Ryerson CJ, Vittinghoff E, Ley B, Lee JS, Mooney JJ, Jones KD, Elicker BM, Wolters PJ, Koth LL, King TE, Collard HR (2014). Predicting survival across chronic interstitial lung disease: the ILD-GAP model. Chest.

[3] Travis WD, Costabel U, Hansell DM, King TE, Lynch DA, Nicholson AG, Ryerson CJ, Ryu JH, Selman M, Wells AU (2013). An official American Thoracic Society/European Respiratory Society statement: update of the international multidisciplinary classification of the idiopathic interstitial pneumonias. Am J Respir Crit Care Med.

[4] Sperandeo M, Varriale A, Sperandeo G, Filabozzi P, Piattelli ML, Carnevale V, Decuzzi M, Vendemiale G (2009). Transthoracic ultrasound in the evaluation of pulmonary fibrosis: our experience. Ultrasound Med Biol.

[5] Volpicelli G, Elbarbary M, Blaivas M, Lichtenstein DA, Mathis G, Kirkpatrick AW, Melniker L, Gargani L, Noble VE, Via G (2012). International evidence-based recommendations for point-of-care lung ultrasound. Intensive Care Med.

[6] Hasan AA, Makhlouf HA (2014). B-lines: transthoracic chest ultrasound signs useful in assessment of interstitial lung diseases. Ann Thorac Med.

[7] Pinal-Fernandez I, Pallisa-Nunez E, Selva-O'Callaghan A, Castella-Fierro E, Simeon-Aznar CP, Fonollosa-Pla V, Vilardell-Tarres M (2015). Pleural irregularity, a new ultrasound sign for the study of interstitial lung disease in systemic sclerosis and antisynthetase syndrome. Clin Exp Rheumatol.

[8] Tardella M, Gutierrez M, Salaffi F, Carotti M, Ariani A, Bertolazzi C, Filippucci E, Grassi W (2012). Ultrasound in the assessment of pulmonary fibrosis in connective tissue disorders: correlation with high-resolution computed tomography. J Rheumatol.

[9] Vizioli L, Ciccarese F, Forti P, Chiesa AM, Giovagnoli M, Mughetti M, Zompatori M, Zoli M (2017). Integrated use of lung ultrasound and chest X-ray in the detection of interstitial lung disease. Respiration.

[10] Buda N, Piskunowicz M, Porzezinska M, Kosiak W, Zdrojewski Z (2016). Lung ultrasonography in the evaluation of interstitial lung disease in systemic connective tissue diseases: criteria and severity of pulmonary fibrosis—analysis of 52 patients. Ultraschall Med.

[11] Zhu WW, Li YD, Li H, Lu XZ, Kong LY, Ye XG, Cai QZ, Sun LL, Jiang W, Wang L (2017). Integrative cardiopulmonary ultrasound for interstitial lung disease assessment: correlation between lung ultrasound performance and cardiac involvement. Ultrasound Med Biol.

[12] Price S, Platz E, Cullen L, Tavazzi G, Christ M, Cowie MR, Maisel AS, Masip J, Miro O, McMurray JJ (2017). Expert consensus document: echocardiography and lung ultrasonography for the assessment and management of acute heart failure. Nat Rev Cardiol.

[13] Andersen CU, Mellemkjaer S, Hilberg O, Nielsen-Kudsk JE, Simonsen U, Bendstrup E (2012). Pulmonary hypertension in interstitial lung disease: prevalence, prognosis and 6 min walk test. Respir Med.

[14] Huston JH, Maron BA, French J, Huang S, Thayer T, Farber-Eger EH, Wells QS, Choudhary G, Hemnes AR, Brittain EL (2019). Association of mild echocardiographic pulmonary hypertension with mortality and right ventricular function. JAMA Cardiol.

[15] American Thoracic S, European Respiratory S: American Thoracic Society/European Respiratory Society International Multidisciplinary Consensus Classification of the Idiopathic Interstitial Pneumonias. This joint statement of the American Thoracic Society (ATS), and the European Respiratory Society (ERS) was adopted by the ATS board of directors, June 2001 and by the ERS Executive Committee, June 2001. Am J Respir Crit Care Med 2002, 165:277–304. 10.1164/ajrccm.165.2.ats01 11790668

[16] Lang RM, Badano LP, Mor-Avi V, Afilalo J, Armstrong A, Ernande L, Flachskampf FA, Foster E, Goldstein SA, Kuznetsova T (2015). Recommendations for cardiac chamber quantification by echocardiography in adults: an update from the American Society of Echocardiography and the European Association of Cardiovascular Imaging. Eur Heart J Cardiovasc Imaging.

[17] Caminati A, Cassandro R, Harari S (2013). Pulmonary hypertension in chronic interstitial lung diseases. Eur Respir Rev.

[18] Jarman ER, Khambata VS, Yun Ye L, Cheung K, Thomas M, Duggan N, Jarai G (2014). A translational preclinical model of interstitial pulmonary fibrosis and pulmonary hypertension: mechanistic pathways driving disease pathophysiology. Physiol Rep.

[19] Chin KM, Kim NH, Rubin LJ (2005). The right ventricle in pulmonary hypertension. Coron Artery Dis.

[20] Rivera-Lebron BN, Forfia PR, Kreider M, Lee JC, Holmes JH, Kawut SM (2013). Echocardiographic and hemodynamic predictors of mortality in idiopathic pulmonary fibrosis. Chest.

[21] Sano H, Tanaka H, Motoji Y, Fukuda Y, Sawa T, Mochizuki Y, Ryo K, Matsumoto K, Emoto N, Hirata K (2015). Right ventricular function and right-heart echocardiographic response to therapy predict long-term outcome in patients with pulmonary hypertension. Can J Cardiol.

[22] D'Andrea A, Stanziola A, Di Palma E, Martino M, D'Alto M, Dellegrottaglie S, Cocchia R, Riegler L, Betancourt Cordido MV, Lanza M (2016). Right ventricular structure and function in idiopathic pulmonary fibrosis with or without pulmonary hypertension. Echocardiography.

[23] Bax S, Jacob J, Ahmed R, Bredy C, Dimopoulos K, Kempny A, Kokosi M, Kier G, Renzoni E, Molyneaux PL (2020). Right ventricular to left ventricular ratio at CT pulmonary angiogram predicts mortality in interstitial lung disease. Chest.

[24] Haddad F, Hunt SA, Rosenthal DN, Murphy DJ (2008). Right ventricular function in cardiovascular disease, part I: anatomy, physiology, aging, and functional assessment of the right ventricle. Circulation.

[25] Moazedi-Fuerst FC, Kielhauser S, Brickmann K, Tripolt N, Meilinger M, Lufti A, Graninger W (2015). Sonographic assessment of interstitial lung disease in patients with rheumatoid arthritis, systemic sclerosis and systemic lupus erythematosus. Clin Exp Rheumatol.

[26] Travis WD, Matsui K, Moss J, Ferrans VJ (2000). Idiopathic nonspecific interstitial pneumonia: prognostic significance of cellular and fibrosing patterns: survival comparison with usual interstitial pneumonia and desquamative interstitial pneumonia. Am J Surg Pathol.

[27] Zhu W, Yidan L, Hong L, Lyu X (2017). Assessment of the correIation between the severity of interstitial lung disease and clinical parameters by cardiopulmonary ultrasound performance. Chin J Ultrason.

[28] Gargani L, Bruni C, Romei C, Frumento P, Moreo A, Agoston G, Guiducci S, Bellando-Randone S, Lepri G, Belloli L (2020). Prognostic value of lung ultrasound B-lines in systemic sclerosis. Chest.

[29] Wells AU, Antoniou KM (2014). The prognostic value of the GAP model in chronic interstitial lung disease: the quest for a staging system. Chest.

[30] Torrisi SE, Ley B, Kreuter M, Wijsenbeek M, Vittinghoff E, Collard HR, Vancheri C (2019). The added value of comorbidities in predicting survival in idiopathic pulmonary fibrosis: a multicentre observational study. Eur Respir J.

